# Gel immersion endoscopic mucosal resection for pedunculated Brunner’s gland hyperplasia in the duodenal bulb near the pylorus

**DOI:** 10.1055/a-2139-4068

**Published:** 2023-08-21

**Authors:** Chihiro Goto, Kenichiro Okimoto, Tomoaki Matsumura, Naoki Akizue, Keisuke Matsusaka, Jun Kato, Naoya Kato

**Affiliations:** 1 Department of Gastroenterology, Graduate School of Medicine, Chiba University, Chiba, Japan; 2 Department of Diagnostic Pathology, Graduate School of Medicine, Chiba University, Chiba, Japan

Brunner’s gland hyperplasia is a benign tumor that occurs primarily in the duodenal bulb. Endoscopic resection is indicated when there is bleeding, obstructive symptoms, or the lesion is large and malignancy cannot be ruled out 1 . Conventional endoscopic mucosal resection (EMR) for the resection of Brunner’s gland hyperplasia has been reported 2 3 . However, conventional EMR of large Burner’s gland hyperplasia in the duodenal bulb is often difficult because the space is narrowed by local injection, reducing visibility and maneuverability. Underwater EMR may be an effective method 4 , but it is often difficult to secure the visual field owing to poor saline retention in the duodenal bulb near the pylorus. Gel immersion EMR (GI-EMR) has recently been reported for duodenal tumors 5 . Here we report the usefulness of GI-EMR in the duodenal bulb near the pylorus for pedunculated Brunner’s gland hyperplasia.

A 30-mm pedunculated submucosal tumor was found in the duodenal bulb near the pylorus ( Fig. 1 a ). As biopsy, computed tomography, and endoscopic ultrasonography could not diagnose the submucosal tumor and its size was large, we decided to perform endoscopic resection. GI-EMR was performed using an upper endoscope (GIF-H290T; Olympus Medical Systems, Tokyo, Japan) and gel (VISCOCLEAR; Otsuka Pharmaceutical Factory, Tokushima, Japan) ( Video 1 ). The retention of the gel in the duodenal bulb was good, even with a small volume (100 mL) ( Fig. 1 b ). No additional gel immersion was required at the time of resection. The gel provided a good field of view and enabled snaring while confirming the origin of the stem ( Fig. 1 c ). Clip closure after resection was also performed partially under gel conditions ( Fig. 1 d ). The pathological finding was Brunner’s gland hyperplasia with no malignancy ( Fig. 1 e ).

**Fig. 1:**
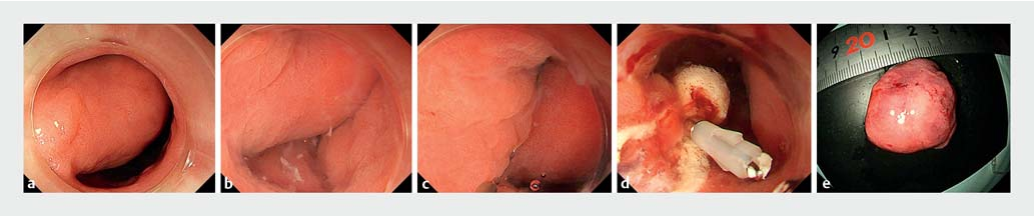
Before and after gel immersion endoscopic mucosal resection (GI-EMR) for the removal of Brunner’s gland hyperplasia. **a** On the day of GI-EMR, a 30-mm pedunculated submucosal tumor of the duodenal bulb was identified near the pylorus. **b** 100 mL of gel filled the lumen and retention was good. **c** The gel in the duodenal bulb made the tumor float, allowing snaring while checking the origin of the stem. **d** The gel filling also facilitated clipping by floating the wound edges. **e** The size of the resected specimen was approximately 30 mm. The pathological finding was Brunner’s gland hyperplasia with no malignancy.

**Video 1** Gel immersion endoscopic mucosal resection for pedunculated Brunner’s gland hyperplasia in the duodenal bulb near the pylorus.


GI-EMR was considered useful for resection of large Brunner’s gland hyperplasia in the duodenal bulb near the pylorus.

Endoscopy_UCTN_Code_TTT_1AO_2AG

## Notes

Endoscopy E-Videos

https://eref.thieme.de/e-videos
*E-Videos* is an open access online section of the journal *Endoscopy* , reporting on interesting cases and new techniques in gastroenterological endoscopy. All papers include a high-quality video and are published with a Creative Commons CC-BY license. Endoscopy E-Videos qualify for HINARI discounts and waivers and eligibility is automatically checked during the submission process. We grant 100% waivers to articles whose corresponding authors are based in Group A countries and 50% waivers to those who are based in Group B countries as classified by Research4Life (see: https://www.research4life.org/access/eligibility/ ). This section has its own submission website at https://mc.manuscriptcentral.com/e-videos
